# Clinical Researches in Electro-Surgery

**Published:** 1874-03

**Authors:** 


*Clinical Researches in Electro-Surgery.* By A. D. Rockwell, A. M. M. D., and George M. Beard, A. M. M. D. New York: Wm. Wood & Co., 1873. Buffalo: H. H. Otis.

This little essay is composed of reports of cases in practice in which electricity in some form was employed.

The authors are well known for their researches in electro-therapeutics, and the present contribution to surgical science will be read with interest by those who are desirous of investigating the practical benefits of electricity.

Much has yet to be observed in order to demonstrate the exact place which is to be allotted to electricity in our list of therapeutical applications. The time has not yet arrived when we can explain its action in all cases in accordance with physiological laws, but careful and patient observation will do much to place it upon a firm basis. Undoubtedly much that is claimed for electricity at present will in time be found to be fallacious, but that its application in many instances is attended with benefit we can see no reason to doubt. Its surgical applications are limited to a certain extent, but there are many instances in which either the use of electrolysis or the galvano-cautery, would be of material aid to the surgeon.

